# Melatonin pulse treatment optimization: boosting vase life and postharvest quality in cut roses (*Rosa hybrida* cv. Samurai)

**DOI:** 10.1186/s12870-026-08911-1

**Published:** 2026-05-09

**Authors:** Sara Shamsinejad, Safoora Saadati, Vahid Reza Saffari, Zahra Pakkish

**Affiliations:** Department of Horticultural Science, Faculty of Agriculture, Shahid Bahonar University of Kerman, P.O. Box: 76169-133, Kerman, Iran

**Keywords:** Melatonin, Postharvest, Roses, Vase life, Quality

## Abstract

Cut roses (*Rosa hybrida* cv. Samurai) exhibit rapid postharvest senescence, limiting vase life and commercial value. This study optimized melatonin application by comparing pulse (30-min immersion) and continuous (vase solution) methods at 0.1 mM and 1 mM concentrations. The 0.1 mM pulse treatment extended vase life by 61% (9.67 vs. 6.00 days in control), improved flower quality score to 5.00, increased flower diameter by 18%, elevated chlorophyll and anthocyanin contents, and reduced electrolyte leakage by 43%. Strong positive correlations (*r* = 0.71–0.94, *P* < 0.001) were observed between these parameters and vase life. Higher concentrations (1 mM) and continuous application were less beneficial. No gray mold symptoms were observed in any treatment during the vase period. These results demonstrate that a brief 0.1 mM melatonin pulse effectively delays senescence and primes endogenous antioxidant defenses, providing one of the first a resource-efficient protocol that warrants further economic and commercial evaluation.

**Supplementary Information:**

The online version contains supplementary material available at 10.1186/s12870-026-08911-1.

## Introduction

Roses (*Rosa* spp.), known as the “queen of flowers”, are central to the global floriculture industry, valued for their aesthetic appeal, color diversity, and cultural significance [1]. The cut flower market exceeded $40 billion in 2024, with roses dominating due to their use in arrangements and gifting [2]. However, postharvest longevity is limited by rapid senescence, causing economic losses and reduced consumer satisfaction [3–5]. This complex deterioration process is driven by an interplay of physiological and biochemical stressors, including: (1) oxidative damage from reactive oxygen species (ROS), which compromises membrane integrity, lipids, and proteins [6, 7]; (2) ethylene-induced petal abscission and senescence via upregulation of biosynthetic genes [8, 9]; (3) vascular occlusions from microbial proliferation and physiological plugging, impeding water uptake [10]; and (4) loss of hydraulic conductivity and turgor pressure, resulting in wilting [6, 9, 11, 12]. Conventional postharvest interventions, such as chemical preservatives (e.g., silver thiosulfate, 8-hydroxyquinoline) and cold storage, mitigate these issues to varying degrees but are limited by inconsistent efficacy, high costs, and environmental concerns from chemical residues [13–16]. This necessitates innovative, sustainable strategies to enhance vase life while aligning with eco-friendly floriculture practices [17, 18].

Melatonin (N-acetyl-5-methoxytryptamine), a pleiotropic indoleamine, has emerged as a promising biostimulant in postharvest horticulture, extending beyond its established role in circadian regulation to exhibit potent antioxidant and regulatory functions in plants [19, 20]. Its efficacy in delaying senescence is well-documented across cut flowers, including roses [21], anthurium [22], carnations [23, 24], gerberas [25], Chrysanthemum [26], and peonies [27], as well as fruits like sweet cherries [28], and pears [29]. Melatonin’s multifaceted mechanisms include: (1) direct scavenging of ROS (e.g., superoxide, hydrogen peroxide) and upregulation of antioxidant enzymes such as superoxide dismutase (SOD), catalase (CAT), ascorbate peroxidase (APX), and glutathione reductase (GR), mitigating oxidative stress [20, 28, 30]; (2) suppression of ethylene biosynthesis through downregulation of ACC synthase (ACS) and ACC oxidase (ACO) genes, reducing petal abscission [29, 31]; (3) enhancement of hydraulic conductivity, improving water relations and turgor maintenance [32–34]; and (4) antimicrobial activity that curbs bacterial growth in vase solutions, preventing vascular blockages [25, 35, 36]. Additionally, melatonin modulates phytohormone crosstalk, elevating indole-3-acetic acid (IAA) to counteract abscisic acid (ABA)-mediated stress and sustaining carbohydrate reserves to support energy demands [37–39]. These actions collectively preserve chlorophyll content, delay petal senescence, and enhance visual quality [40, 41].

Despite these documented benefits, the optimal delivery method for melatonin in cut rose preservation remains ambiguous. Among the available application strategies, pulse treatment offers distinct advantages. Pulse treatment is defined as a short-term immersion of stem bases in a melatonin solution followed by transfer to a preservative free vase solution. This approach provides resource efficiency and minimized chemical exposure. Conversely, continuous application keeps melatonin in the vase solution throughout the postharvest period. This method may provide sustained protection but carries the risk of diminished returns due to potential receptor desensitization or microbial degradation. The efficacy of pulse-based protocols has been corroborated in other ornamental species. For instance, a citric acid pulse significantly prolonged gerbera vase life through enhanced water relations [42]. Likewise, a melatonin pulse delayed the senescence process in cut *Ranunculus asiaticus* flowers, extended vase life to more than 18 days, maintained higher leaf chlorophyll content, improved water balance, and preserved photosynthetic performance compared to untreated controls [43].

Nevertheless, within the context of cut roses, a systematic comparison between pulse and continuous melatonin application remains conspicuously absent, particularly regarding the interaction of application method with concentration [21, 23]. Specifically, whether a brief melatonin pulse outperforms continuous exposure and whether lower concentrations such as 0.1 mM suffice relative to higher ones such as 1 mM has yet to be determined.

While previous studies have demonstrated the efficacy of melatonin in extending vase life of various cut flowers [21–27], a critical knowledge gap remains: the comparative effectiveness of pulse versus continuous application methods, and their interaction with concentration, has not been systematically evaluated in cut roses. Furthermore, most existing studies have used continuous application in vase solutions, which may be less resource-efficient than a brief pulse treatment. The present study fills this gap by directly comparing pulse (30-min immersion) and continuous (vase solution) methods at two distinct concentrations (0.1 and 1 mM), thereby providing the first systematic evaluation of application method × concentration interactions in rose postharvest physiology.

This study differs from prior work on melatonin in roses [21] in three key aspects: (1) it provides the first direct comparison of pulse versus continuous application methods; (2) it systematically evaluates the interaction between application method and concentration (0.1 vs. 1 mM); and (3) it tests the hypothesis that a brief pulse acts as a priming stimulus rather than a sustained preservative. The concentrations used (0.1 mM and 1 mM) were based on previous reports demonstrating efficacy in related species [21, 23], while the 30-minute pulse duration was chosen as a commercially feasible short-term treatment that minimizes chemical usage and labor requirements compared to continuous application.

Accordingly, we hypothesized that a short-term melatonin pulse acts as a priming cue. This priming cue elicits a more robust and sustained activation of endogenous antioxidant and water relation pathways compared to continuous exposure. Furthermore, this response was hypothesized to be concentration dependent. To test this hypothesis, the present study was designed to evaluate the effects of pulse versus continuous melatonin treatments at 0.1 mM and 1 mM concentrations on vase life, floral quality, physiological integrity, and antioxidant enzyme activities of cut roses (*Rosa hybrida* L. cv. Samurai). The overarching goal was to establish an optimized melatonin application protocol that could serve as a foundation for future economic and commercial evaluations.

## Materials and methods

### Plant material

Cut roses (*Rosa hybrida* L. cv. Samurai) were harvested at the commercial maturity stage (tight bud, outer petals unfurling) from a commercial greenhouse in Zarand, Kerman province, Iran. Stems were uniformly selected for length (approximately 50 cm), free from defects or diseases, and immediately transported to the laboratory under hydrated conditions (wrapped in moist paper and placed in buckets with distilled water). Upon arrival, stems were re-cut underwater to a uniform length of 40 cm to remove air emboli, basal leaves were removed to prevent microbial contamination in vase solutions, and 4 leaves were retained per stem to standardize foliage load and minimize variability in physiological measurements. Each treatment was replicated three times, with each replicate consisting of 10 cut stems (one stem per vase), resulting in a total of 150 stems (5 treatments × 3 replicates × 10 stems).

### Preparation of melatonin solutions

Melatonin (N-acetyl-5-methoxytryptamine, purity ≥ 98%, Sigma-Aldrich, USA) was dissolved in absolute ethanol (0.1% v/v) as a co-solvent to enhance solubility, then diluted to the desired concentrations (0.1 mM and 1 mM) using distilled water. Control solutions consisted of distilled water with 0.1% ethanol to account for any solvent effects. All solutions were freshly prepared on the day of treatment and adjusted to pH 5.5–6.0 using 0.1 M HCl or NaOH to mimic typical vase water conditions [12].

### Treatment applications

The experiment followed a completely randomized design (CRD) with a 2 × 2 factorial arrangement (method: pulse/continuous; concentration: 0.1/1 mM) plus a control, resulting in five treatments: (1) control (distilled water with 0.1% ethanol), (2) 0.1 mM melatonin pulse, (3) 1 mM melatonin pulse, (4) 0.1 mM melatonin continuous, and (5) 1 mM melatonin continuous. All treatments were applied immediately after stem preparation. For pulse treatments, stems were immersed up to 10 cm from the base in melatonin solutions (0.1 or 1 mM) for 30 min at 20 °C in the dark, then rinsed with distilled water and transferred to vases with fresh distilled water, refreshed every 48 h. For continuous treatments, stems were placed in vases with melatonin-infused solutions (0.1 or 1 mM), replaced every 48 h to maintain concentration. To prevent melatonin degradation under light, all vases were covered with opaque material [19]. Vases were maintained at 20 ± 2 °C, 60–70% relative humidity, and a 12-hour photoperiod (15–20 µmol m⁻² s⁻¹ PPFD). Each treatment had three replicates of 10 stems (one per vase).

### Measurement of postharvest parameters

Postharvest parameters were assessed primarily at the conclusion of the vase life period, with the exception of vase life, flower quality, and solution uptake, which were evaluated dynamically throughout the experiment. Physiological and biochemical parameters were measured on leaf tissues, while anthocyanin content was quantified specifically in petals.

### Vase life

Vase life was determined as the number of days from the start of the experiment until flowers exhibited senescence, defined as wilting of > 50% of petals, petal discoloration, or bent neck (peduncle bending > 45°). Daily visual assessments were conducted by two independent observers, and the average was recorded [44, 45].

### Flower quality

Flower quality was evaluated daily on a subjective scale from 1 to 5, where: 5 = excellent (fully turgid, vibrant color, no wilting); 4 = good (minor petal edge curling, slight color fading); 3 = moderate (noticeable wilting on 25–50% petals); 2 = poor (severe wilting, significant discoloration); and 1 = unacceptable (complete wilting or abscission). Scores were averaged across replicates [46].

### Flower diameter

Flower diameter was measured using a digital caliper (accuracy ± 0.01 mm) at the end of the vase life period. The maximum diameter of the flower head was recorded as the distance across the outermost petals at their widest point. Measurements were taken in two perpendicular directions, and the average was calculated for each flower [47].

### Chlorophyll content index

Chlorophyll content index was indirectly assessed as the chlorophyll index using a SPAD-502 chlorophyll meter (Konica Minolta, Japan) at the end of the vase life period. Measurements were taken on the middle portion of three leaves per stem, and the average SPAD value was recorded [48].

### Pigment content (chlorophyll, carotenoids, and anthocyanins)

Fresh leaf tissue was pulverized in 70% acetone and centrifuged at 350 × g for 15 min at ambient temperature to produce a clarified supernatant. The absorbance of this extract was recorded spectrophotometrically at 663.2 nm, 646.8 nm, and 470 nm. Pigment levels were determined using equations derived from Lichtenthaler [49] and expressed as mg g⁻¹ fresh weight (FW).

Anthocyanin content was measured in petal samples collected at the end of the vase life period. A 0.5 g sample of fresh petal tissue was extracted in 10 mL of acidified methanol (1% HCl v/v) overnight at 4 °C in the dark. After centrifugation (10,000 × g, 10 min), absorbance was measured at 530 nm and 657 nm using a spectrophotometer [50].

### Electrolyte leakage

Electrolyte leakage, an indicator of membrane integrity, was measured using leaf discs (1 cm diameter, 10 discs per replicate) excised at the end of the vase life period. Discs were washed with distilled water, immersed in 20 mL distilled water, and shaken at 100 rpm for 24 h at 25 °C. Initial conductivity (EC_1_) was measured using a conductivity meter (Apera EC20, Apera Instruments, China). Samples were then autoclaved at 121 °C for 20 min to release total electrolytes, cooled, and final conductivity (EC_2_) was measured. Ion leakage (%) was calculated as: (EC_1_ / EC_2_) × 100 [51].

### Relative Water Content (RWC)

Relative water content was assessed on leaf samples collected at the end of the vase life period. Fresh weight (F_W_) was recorded immediately after sampling. Leaves were floated in distilled water for 24 h at 4 °C to achieve turgid weight (T_W_), then oven-dried at 70 °C for 48 h to obtain dry weight (D_W_). RWC (%) was calculated as: [(F_W_ – D_W_) / (T_W_ – D_W_)] × 100 [52].

### Solution uptake

Solution uptake was measured daily by recording the volume of solution consumed per stem. Vases were initially filled with 500 mL of treatment solution or distilled water (control). Each day, the volume of solution remaining in each vase was measured, and the consumed volume was replenished to maintain 500 mL. The total volume consumed per stem was calculated as the sum of daily consumption over the vase life period, adjusted for evaporative losses using control vases without stems. Solution uptake was expressed as mL stem⁻¹ and averaged across replicates [53].

### Fresh-to-dry weight ratio

The fresh-to-dry weight ratio was determined to assess the plant’s hydration status at the end of the vase life period. Leaf samples were collected and immediately weighed to obtain the fresh weight (F_W_). The samples were then oven-dried at 70 °C for 48 h until a constant dry weight (D_W_) was achieved. The fresh-to-dry weight ratio was calculated using the formula F_W_ / D_W_ [54].

### Antioxidant enzyme activities

Peroxidase (POD) and catalase (CAT) activities were measured using leaf samples collected at the end of the vase life period. For both enzymes, 0.5 g of fresh leaf tissue was homogenized in 5 mL of 50 mM phosphate buffer (pH 7.0) containing 1% polyvinylpyrrolidone (PVP) at 4 °C, centrifuged at 12,000 × g for 15 min at 4 °C, and the supernatant used as the enzyme extract.

POD activity was determined by measuring guaiacol oxidation at 470 nm (UV-1800, Shimadzu, Japan) in a reaction mixture containing 50 mM phosphate buffer (pH 7.0), 20 mM guaiacol, 10 mM H₂O₂, and 0.1 mL enzyme extract. Activity was expressed as U g⁻¹ FW, where one unit (U) represents the amount of enzyme causing a 0.01 increase in absorbance per minute [55].

CAT activity was measured by monitoring H₂O₂ decomposition at 240 nm in a reaction mixture containing 50 mM phosphate buffer (pH 7.0), 15 mM H₂O₂, and 0.1 mL enzyme extract. Activity was expressed as U g⁻¹ FW, where one unit (U) represents the amount of enzyme causing a 0.01 increase in absorbance per minute, calculated using the extinction coefficient of 39.4 mM⁻¹ cm⁻¹ [56].

### Statistical analysis

Data were analyzed using analysis of variance (ANOVA) in SAS software (version 9.4, SAS Institute, USA). The factorial arrangement included two factors: application method (pulse vs. continuous) and concentration (0.1 mM vs. 1 mM), with a control treatment. Means were separated using Duncan’s multiple range test at *P* ≤ 0.05.

## Results

The effects of melatonin applied via pulsed or continuous methods at 0.1 mM and 1 mM concentrations on postharvest characteristics of cut rose flowers were evaluated against an untreated control. All measured parameters, except carotenoid content, exhibited statistically significant differences (*p* < 0.05).

### Vase life and overall quality

**Fig. 1:**
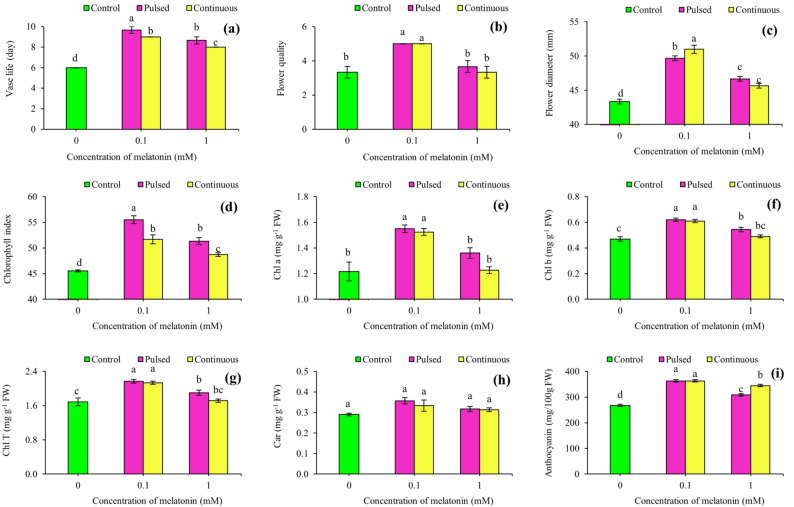
Effects of melatonin treatments (pulse and continuous) at different concentrations of melatonin (mM) on postharvest parameters of cut roses (*Rosa hybrida* L. cv. Samurai), including vase life (**a**), flower quality (**b**), flower diameter (**c**), chlorophyll index (**d**), chlorophyll a (**e**) chlorophyll b (**f**) total chlorophyll (**g**), carotenoid (**h**), anthocyanin (**i**) contents. Bars represent means ± SE (*n* = 3), with different letters indicating significant differences (Duncan’s test, *P* ≤ 0.05)

The control group had an average vase life of 6.00 days. Melatonin treatments extended vase life, with pulsed 0.1 mM melatonin, achieving the longest duration at 9.67 days (a 61% increase), followed by continuous 0.1 mM melatonin at 9.00 days (a 50% increase). The 1 mM treatments were less effective, with vase lives of 8.67 days for pulsed and 8.00 days for continuous applications (Fig. 1a). Flower quality, rated on a 1–5 scale, averaged 3.33 in the control. Both pulsed and continuous 0.1 mM melatonin treatments achieved the maximum score of 5.00, while 1 mM treatments yielded lower scores of 3.67 for pulsed and 3.33 for continuous applications (Fig. 1b). Correlation analysis revealed a strong positive association between vase life and flower quality (*r* = 0.71, *p* < 0.01), indicating that enhanced quality contributes significantly to prolonged longevity (Table 1).

**Table 1:** Pearson correlation coefficients among postharvest parameters of cut roses (*Rosa hybrida* L. cv. Samurai) treated with melatonin

	VF	FQ	FD	CI	Chl a	Chl b	Chl T	Car	Ant	EL	RWC	SU	FW/FD	POD	CAT
VF	1														
FQ	0.71 ^**^	1													
FD	0.84 ^***^	0.82 ^***^	1												
CI	0.90 ^***^	0.75 ^**^	0.77 ^**^	1											
Chl a	0.76 ^***^	0.84 ^***^	0.88 ^***^	0.74 ^**^	1										
Chl b	0.81 ^***^	0.82 ^***^	0.91 ^***^	0.78 ^***^	0.98 ^***^	1									
Chl T	0.78 ^***^	0.84 ^***^	0.87 ^***^	0.76 ^**^	0.99 ^***^	0.99 ^***^	1								
Car	0.58 ^*^	0.48 ^ns^	0.68 ^**^	0.60 ^*^	0.62 ^*^	0.66 ^**^	0.64 ^**^	1							
Ant	0.81 ^***^	0.67 ^**^	0.84 ^***^	0.74 ^**^	0.65 ^**^	0.70 ^**^	0.67 ^**^	0.64 ^**^	1						
EL	-0.71 ^**^	-0.61 ^*^	-0.88 ^***^	-0.59 ^*^	-0.65 ^**^	-0.74 ^**^	-0.68 ^**^	-0.50 ^*^	-0.70 ^**^	1					
RWC	0.83^***^	0.87 ^***^	0.93 ^***^	0.87 ^***^	0.88 ^***^	0.92 ^***^	0.89 ^***^	0.60 ^*^	0.81 ^***^	-0.79 ^***^	1				
SU	0.80 ^***^	0.71 ^**^	0.84 ^***^	0.80 ^***^	0.74 ^**^	0.78 ^***^	0.75 ^**^	0.72 ^**^	0.85 ^***^	-0.777 ^***^	0.85 ^***^	1			
FW/FD	0.86 ^***^	0.76 ^***^	0.86 ^***^	0.91 ^***^	0.78 ^**^	0.83 ^***^	0.80 ^***^	0.74 ^**^	0.85 ^***^	-0.75 ^**^	0.92 ^***^	0.93 ^***^	1		
POD	0.94 ^***^	0.70 ^**^	0.89 ^***^	0.86 ^***^	0.78 ^***^	0.83 ^***^	0.80 ^***^	0.69 ^**^	0.93 ^***^	-0.75 ^**^	0.87 ^***^	0.88 ^***^	0.91 ^***^	1	
CAT	0.92 ^***^	0.74 ^**^	0.88 ^***^	0.89 ^***^	0.82 ^***^	0.86 ^***^	0.83 ^***^	0.66 ^**^	0.76 ^***^	-0.77 ^**^	0.87 ^***^	0.88 ^***^	0.91 ^***^	0.90 ^***^	1

Significance levels are denoted as: * (*P* < 0.05), ** (*P* < 0.01), *** (*P* < 0.001), ns (non-significant, *P* > 0.05)

*VF* Vase life, *FQ *Flower quality, *FD *flower diameter, *CI *Chlorophyll index, *Chl a *Chlorophyll a, *Chl b *Chlorophyll b, *Chl T *Chlorophyll T, *Car *Carotenoids, *Ant *Anthocyanins, *EL *Electrolyte leakage, *RWC *Relative water content, *SU *Solution uptake, *FW/FD *Fresh-to-dry weight ratio, *c *Peroxidase activity, *CAT *Catalase activity

Daily monitoring of senescence symptoms revealed distinct patterns among treatments. Control flowers showed initial signs of senescence (minor petal edge curling and slight color fading) on day 4. By day 5, control flowers exhibited petal wilting exceeding 50%, noticeable discoloration, and bent neck (peduncle bending > 45°), leading to termination of vase life by day 6. In contrast, flowers treated with 0.1 mM melatonin (both pulse and continuous) maintained full turgor, vibrant color, and normal peduncle orientation until day 8, with only minor petal edge curling observed thereafter. The 0.1 mM pulse treatment retained acceptable ornamental quality until day 9.67. The 1 mM treatments showed intermediate symptoms, with noticeable wilting appearing around day 6 and bent neck observed by day 8 for continuous application. No gray mold (*Botrytis cinerea*) symptoms were observed in any treatment during the entire vase period.

### Morphological and pigment-related parameters

The control group exhibited an average flower diameter of 43.33 mm. Melatonin treatments increased flower diameter, with continuous 0.1 mM melatonin yielding the highest value at 51.00 mm (an 18% increase), followed by pulsed 0.1 mM melatonin at 49.67 mm. The 1 mM treatments resulted in intermediate diameters of 46.67 mm for pulsed and 45.67 mm for continuous applications (Fig. 1c). Flower diameter showed a strong positive correlation with vase life (*r* = 0.84, *p* < 0.001), highlighting its role in extending postharvest longevity (Table 1).

The chlorophyll index in the control group was 45.50, with pulsed 0.1 mM melatonin showing the highest increase of 22%, followed by continuous 0.1 mM melatonin at approximately 14%. The 1 mM treatments exhibited smaller gains: about 13% for pulsed and 7% for continuous applications (Fig. 1d). Similarly, chlorophyll contents in the control were Chl a: 1.22 mg g⁻¹ FW, Chl b: 0.47 mg g⁻¹ FW, and total: 1.69 mg g⁻¹ FW. Pulsed 0.1 mM melatonin yielded the greatest enhancements—Chl a by roughly 27%, Chl b by 32%, and total by 28%—while continuous 0.1 mM melatonin followed closely with increases of about 25% for Chl a, 30% for Chl b, and 26% for total. The 1 mM treatments showed more modest improvements: pulsed with around 11% for Chl a, 15% for Chl b, and 12% for total; and continuous with minimal rises of 1% for Chl a, 4% for Chl b, and 2% for total (Fig. 1e-g). Overall, chlorophyll content displayed a strong positive correlation with vase life (*r* > 0.75, *p* < 0.01), highlighting its role in extending flower longevity (Table 1).

Carotenoid content in the control was 0.29 mg g⁻¹ FW, with slight increases for pulsed and continuous 0.1 mM melatonin treatments, and even smaller changes for 1 mM treatments. These differences were not statistically significant (Fig. 1h).

Anthocyanin content in the control group was measured at 267.41 mg/100 g fresh weight (FW). Application of 0.1 mM melatonin, both pulsed and continuous, significantly elevated anthocyanin levels by approximately 36%, reaching 362.97 mg/100 g FW. In contrast, 1 mM melatonin treatments resulted in more moderate increases, with pulsed application achieving 308.43 mg/100 g FW (15% increase) and continuous application reaching 344.88 mg/100 g FW (29% increase) (Fig. 1i). Statistical analysis demonstrated a strong positive correlation between anthocyanin content and vase life (*r* = 0.81, *p* < 0.001), highlighting its pivotal role in enhancing color vibrancy and extending flower longevity (Table 1).

### Physiological and water relations parameters

**Fig. 2:**
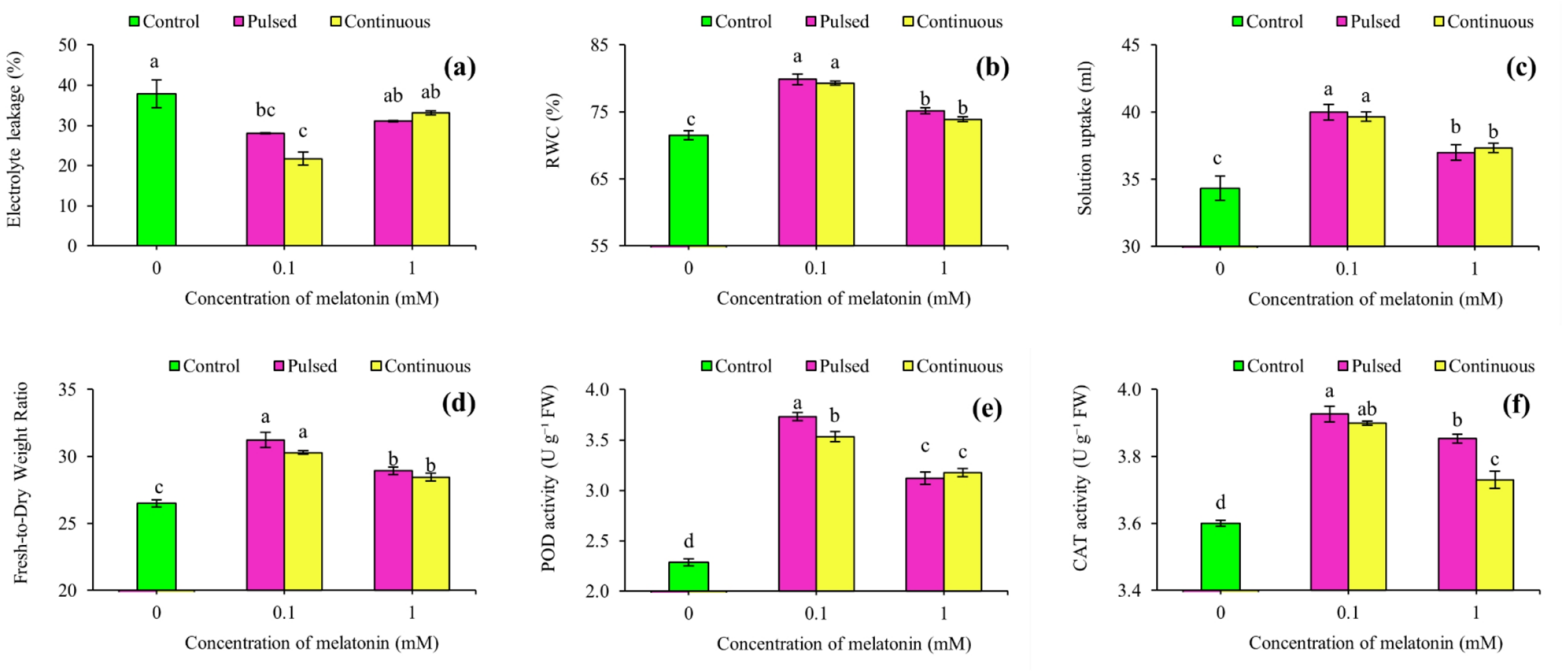
Effects of melatonin treatments (pulse and continuous) at different concentrations of melatonin (mM) on postharvest parameters of cut roses (*Rosa hybrida* L. cv. Samurai), including electrolyte leakage (**a**), relative water content (**b**), solution uptake (**c**), fresh-to-dry weight ratio (**d**), peroxidase (**e**) and catalase (**f**) activities. Bars represent means ± SE (*n* = 3), with different letters indicating significant differences (Duncan’s test, *P* ≤ 0.05)

Electrolyte leakage in the control group was measured at 37.81%. Melatonin treatments significantly reduced leakage, with continuous 0.1 mM melatonin achieving the greatest reduction to 21.72%, a 43% decrease, followed by pulsed 0.1 mM at 28.03%, a 26% decrease. The 1 mM treatments showed milder reductions, with 31.05% for pulsed, 18% lower, and 33.10% for continuous, 12% lower (Fig. 2a). A strong negative correlation with vase life was evident (*r* = − 0.71, *p* < 0.01), highlighting the importance of minimizing membrane damage for enhanced postharvest flower preservation (Table 1).

Relative water content (RWC) in the control group averaged 71.84%. Treatment with 0.1 mM melatonin markedly increased RWC to 79.89% for pulsed application, an 11% rise, and 79.32% for continuous application, a 10% rise. The 1 mM treatments yielded smaller increases, reaching 75.20% for pulsed, a 5% rise, and 73.89% for continuous, a 3% rise (Fig. 2b).

Solution uptake in the control samples was 34.33 mL. Melatonin treatments enhanced uptake, with pulsed 0.1 mM reaching 40.00 mL, a 16% increase, and continuous 0.1 mM reaching 39.67 mL, a 15% increase. The 1 mM treatments showed modest gains, with 37.00 mL for pulsed, an 8% increase, and 37.33 mL for continuous, a 9% increase (Fig. 2c).

The fresh-to-dry weight ratio in the control group was recorded at 26.51. Application of 0.1 mM melatonin significantly raised this ratio to 31.25 for pulsed treatment, an 18% increase, and 30.29 for continuous treatment, a 14% increase. The 1 mM treatments resulted in smaller increments, reaching 28.94 for pulsed, a 9% increase, and 28.46 for continuous, a 7% increase (Fig. 2d). Strong positive correlations were observed between vase life and relative water content, solution uptake, and fresh-to-dry weight ratio (*r* > 0.8, *p* < 0.001), emphasizing their critical roles in sustaining hydration and prolonging postharvest flower longevity (Table 1).

### Antioxidant enzyme activities

Peroxidase (POD) activity in the control group was 2.29 U g⁻¹ FW, rising to 3.73 U g⁻¹ FW with pulsed 0.1 mM melatonin, a 63% increase, and 3.53 U g⁻¹ FW with continuous 0.1 mM melatonin, a 54% increase. The 1 mM treatments showed smaller rises, reaching 3.12 U g⁻¹ FW for pulsed, a 36% increase, and 3.18 U g⁻¹ FW for continuous, a 39% increase (Fig. 2e). Catalase (CAT) activity in the control was 3.60 U g⁻¹ FW, increasing to 3.93 U g⁻¹ FW with pulsed 0.1 mM melatonin, a 9% rise, and 3.90 U g⁻¹ FW with continuous 0.1 mM melatonin, an 8% rise. The 1 mM treatments reached 3.85 U g⁻¹ FW for pulsed, a 7% rise, and 3.73 U g⁻¹ FW for continuous, a 4% rise (Fig. 2f). Both POD and CAT activities exhibited strong positive correlations with vase life (*r* > 0.9, *p* < 0.001), underscoring their critical role in mitigating oxidative stress and extending flower longevity (Table 1).

## Discussion

The findings of this study provide compelling evidence that the exogenous application of melatonin, applied either as a pulse or in continuous form at concentrations of 0.1 and 1 mM, exerts profound positive effects on a wide range of postharvest attributes in cut rose flowers, with the exception of carotenoid content, which remained unaffected. Melatonin extended vase life and enhanced flower quality, with 0.1 mM pulsed treatment providing the greatest extension in vase life (9.67 days), and both 0.1 mM treatments achieving maximum quality scores (Fig. 1a-b). A strong positive correlation existed between vase life and flower quality (*r* = 0.71, *p* < 0.01; Table 1). These results complement and extend existing literature: for instance, Mazrou et al. [21] reported that 0.2 mM melatonin nearly doubled the vase life of cut roses by improving RWC, reducing membrane damage, and delaying senescence. Similarly, Lezoul et al. [23] demonstrated that postharvest melatonin application at 0.1 mM lengthened carnation vase life by up to 10 days. Preharvest applications in tuberose (*Polianthes tuberosa*) were also shown to promote water balance and antioxidant activity, further extending floral longevity [57]. Comparable effects have also been reported in peony (*Paeonia lactiflora*), where melatonin delayed senescence by protecting chlorophyll molecules, reducing proline accumulation, and alleviating oxidative stress [27].

While direct comparison with commercial preservatives such as Chrysal, silver thiosulfate, or 8-hydroxyquinoline was beyond the scope of this study, the 0.1 mM melatonin pulse treatment (61% vase life extension) showed efficacy comparable to many reported values for these commercial products in cut roses [13–16]. Future studies directly comparing melatonin pulse treatment with standard commercial preservatives under identical conditions would be valuable. Although concentration (0.1 mM vs. 1 mM) had a more pronounced effect on vase life than application method, the pulse treatment offered additional advantages in terms of reduced chemical usage and labor requirements, making it a more resource-efficient option for commercial application.

At the mechanistic level, the beneficial role of melatonin can be understood from its dual capacity as both a direct ROS scavenger and a modulator of plant hormonal pathways. Melatonin neutralizes reactive oxygen species such as superoxide radicals, hydrogen peroxide, and hydroxyl radicals, thereby protecting vital biomolecules like lipids, proteins, and nucleic acids from oxidative damage. Regarding hormonal regulation, it has been widely reported that melatonin can suppress ethylene biosynthesis and action in various plant species [29, 31]. Nevertheless, the present study did not measure ethylene production rates or the expression of ethylene-related genes. Consequently, while the delay in senescence observed in our melatonin-treated roses is consistent with known anti-ethylene effects of melatonin, any conclusion about ethylene suppression remains speculative at this stage and requires further targeted investigation. Melatonin has also been reported to modulate auxin-related activities that promote tissue vitality and delay senescence in other species [38], though this mechanism was not directly investigated in the present study. Moreover, melatonin has been shown to enhance the transcription of stress-related genes, including those encoding antioxidant enzymes, thereby strengthening cellular protection systems against oxidative stress [20, 26]. This set of interconnected actions explains why melatonin-treated flowers not only live longer but also maintain superior visual and quality attributes.

A key finding of this study is the clear superiority of the 0.1 mM pulse treatment [21, 43]. We propose this is a classic example of hormetic priming. A brief, concentrated exposure to melatonin likely acts as a mild eustress signal, activating the flower’s transcriptional defense machinery more potently than a continuous, low-level stimulus [58, 59]. This ‘primed’ state then persists throughout the vase life, providing sustained protection against senescence triggers. In contrast, continuous exposure, particularly at the higher 1 mM concentration, could lead to receptor desensitization or negative feedback inhibition, diminishing its efficacy over time [60]. This priming hypothesis is consistent with observations in other plant systems where brief stress applications confer long-term resistance [61, 62].

A clear and significant enhancement in flower diameter was also observed under 0.1 mM melatonin, with the continuous treatment producing the largest flowers (51.00 mm, or an 18% increase) (Fig. 1c). Vase life strongly correlated with flower size (*r* = 0.84, *p* < 0.001; Table 1), suggesting that larger flowers are more likely to remain viable for longer when treated with melatonin. Parallel results have been reported by Wang et al. [27], where peonies maintained greater flower size under melatonin treatment due to enhanced water balance, membrane stability, and osmotic regulation. Cut chrysanthemums treated with 5 µM melatonin also displayed increased diameter, fresh weight, and water retention compared to untreated controls [27]. Similarly, marigold plants sprayed with 150 mg/L melatonin exhibited significant increases in both fresh and dry flower weights as well as yield, mainly attributed to improved morpho-physiological processes [63]. From a physiological perspective, melatonin contributes to enlarged flower diameter by promoting cell expansion and division, processes mediated in part by its interactions with auxin and gibberellin signaling pathways. The improvement in water relations parameters—including higher relative water content, increased solution uptake, and enhanced fresh-to-dry weight ratio—suggests that melatonin treatment positively influenced hydraulic conductivity and tissue hydration in cut roses. Similar beneficial effects of melatonin on water uptake and maintenance of plant water status have been reported in other species [64–66]. The observed increase in flower diameter is likely driven by enhanced cell expansion, which may be linked to melatonin’s documented role in improving plant water relations and maintaining cell turgor [36, 67].

Chlorophyll content (including indices for chlorophyll a, chlorophyll b, and total chlorophyll) was significantly elevated by melatonin treatments, with the 0.1 mM pulse treatment producing the highest increases (22% for the chlorophyll index and ~ 28% for total chlorophyll) (Fig. 1d–g). Chlorophyll levels correlated strongly with vase life (*r* > 0.75, *p* < 0.01; Table 1), highlighting their vital role in sustaining postharvest floral metabolism. Similar findings have been observed in chrysanthemums, where melatonin delayed chlorophyll degradation and preserved higher pigment content during storage [26]. Mechanistically, melatonin exerts its chlorophyll-protective effect by stabilizing chloroplast membranes, preventing ROS-mediated damage, and inhibiting chlorophyllase—the enzyme responsible for chlorophyll breakdown [68]. Similar protective effects on chlorophyll content have been reported in other plant species following melatonin treatment [69–71]. Interestingly, carotenoids remained unaffected in this study (Fig. 1h), suggesting that while melatonin selectively regulates chlorophyll metabolism, carotenoid biosynthesis may not be as responsive to melatonin or may be less sensitive to oxidative stress, as reported by Xu et al. [72].

Anthocyanin concentrations also increased substantially under melatonin, particularly in 0.1 mM treatments (~ 36%), compared to 15–29% increases under 1 mM (Fig. 1i). Vase life correlated strongly with anthocyanin levels (*r* = 0.81, *p* < 0.001), indicating that color retention is closely aligned with floral longevity. Anthocyanin content in petals was significantly enhanced by the 0.1 mM melatonin treatments (approximately 36% higher than the control). This increase contributed to better color retention and improved visual quality of the flowers. Similar beneficial effects of melatonin on anthocyanin levels and petal coloration have been reported in other plant species [71, 72].

Electrolyte leakage, a key indicator of membrane integrity, decreased significantly under melatonin treatments, with continuous 0.1 mM yielding the most pronounced reduction (43%) (Fig. 2a). A negative correlation was observed with vase life (*r* = − 0.71, *p* < 0.01), highlighting the value of membrane stability for prolonging floral freshness. Similar outcomes have been observed in cut roses and carnations, where melatonin reduced leakage by enhancing ROS detoxification and preserving lipid bilayer structures [73, 74]. Mechanistically, melatonin can directly integrate into lipid membranes, reinforcing their stability, while also activating enzymatic antioxidants to suppress lipid peroxidation [75, 76]. This reduction in ion leakage provides direct evidence of melatonin’s role in preserving membrane stability. The significant upregulation of POD and CAT activities (Fig. 2e-f) offers a clear enzymatic mechanism for this protection, as these enzymes detoxify H₂O₂ and other ROS, preventing the lipid peroxidation that compromises membrane integrity [77].

Hydration-related traits, such as relative water content (RWC), solution uptake, and fresh-to-dry weight ratios, improved markedly with melatonin, with pulsed 0.1 mM performing slightly better than continuous application (Fig. 2b–d). All hydration parameters correlated strongly with vase life (*r* > 0.8, *p* < 0.001). These effects resonate with findings in tomato, where melatonin sprays increased RWC under heat and drought stress [78]. Hosseini et al. [79] also reported that melatonin extended postharvest longevity in roses and carnations by improving water retention and reducing ROS damage. Previous studies have suggested that melatonin may facilitate water flow through cellular membranes [67, 80]. By modulating stomatal closure, melatonin also optimizes transpiration rates, maintaining turgor pressure and preventing desiccation [81, 82]. Studies also show melatonin maintains partially open stomata under drought stress, balancing transpiration and water retention [82, 83]. Furthermore, melatonin improves biomass accumulation by enhancing photosynthesis and carbohydrate translocation, increasing fresh and dry weights in plants like grapefruit mint and cotton [84, 85]. In petal tissues, melatonin reduces fresh and dry weight loss under drought, maintaining tissue hydration [86]. These mechanisms collectively enhance water status, biomass, and postharvest longevity in flowers.

Finally, antioxidant enzyme activities—specifically POD and CAT—increased significantly under melatonin, with 0.1 mM pulse showing the greatest enhancement (POD by 63%) (Fig. 2e–f). These enzymes exhibited strong correlations with vase life (*r* > 0.8, *p* < 0.001). Such increases mirror other reports in *Ranunculus asiaticus* [43], mung bean [87], and roses [21], supporting melatonin’s strong ROS-scavenging roles. Beyond direct enzymatic upregulation, melatonin also promotes accumulation of non-enzymatic antioxidants such as glutathione and ascorbate [88, 89]. Altogether, these multifaceted defense mechanisms protect membranes from peroxidation, maintain redox balance, and delay senescence [90, 91].

It is important to acknowledge the limitations of this study. First, our conclusions regarding molecular mechanisms (e.g., improved water transport, ethylene suppression) are based on logical inference from phenotypic data, as we did not measure ethylene production, microbial populations, aquaporin gene expression, or antioxidant markers such as SOD, APX, MDA, and total antioxidant capacity (TAC). Second, physiological measurements were taken only at the end of the vase period (not dynamically) and were conducted on leaf tissues rather than petals, which would more directly reflect floral senescence. Third, only two melatonin concentrations were tested, and the study was conducted on a single rose cultivar, limiting generalizability. Fourth, floral senescence assessment relied on anthocyanin content, flower diameter, and subjective scores, without additional objective indicators such as petal turgor pressure or cell wall integrity markers. Finally, no formal cost analysis, labor assessment, or commercial scaling projection was performed, and photographic documentation was not captured. Future studies should address these gaps through time-course sampling, petal-specific analyses, broader dose ranges, multi-cultivar validation, comprehensive antioxidant profiling, economic evaluations, and visual documentation. Finally, no formal cost-benefit analysis, labor cost assessment, or commercial scalability projection was performed, and photographic documentation was not captured.

## Conclusion

This study identifies a 0.1 mM melatonin pulse treatment as an effective strategy to significantly extend the vase life of ‘Samurai’ roses. Although concentration had a more pronounced effect on vase life than application method, the pulse method offers additional advantages as a priming agent, activating the flower’s own defense systems with reduced chemical usage and labor requirements compared to continuous application. Melatonin effectively mitigated major senescence triggers, including ROS accumulation, membrane damage, and water imbalance. The pulse 0.1 mM treatment should be further evaluated for economic feasibility and scalability before commercial recommendation. This foundational work paves the way for future studies to explore the molecular basis of melatonin’s priming effect, further refining its application for sustainable floriculture.

## Supplementary Information

Supplementary Material 1.

## Data Availability

All data supporting the findings of this study are available within the paper and its Supplementary Information.
